# Analysis of the Protective Efficacy of Approved COVID-19 Vaccines Against Various Mutants

**DOI:** 10.3389/fimmu.2022.804945

**Published:** 2022-04-28

**Authors:** Chaonan Li, Yikai Guo, Zhongbiao Fang, Haiyan Zhang, Yanjun Zhang, Keda Chen

**Affiliations:** ^1^ Shulan International Medical College, Zhejiang Shuren University, Hangzhou, China; ^2^ Zhejiang Shuren College, Zhejiang Chinese Medical University, Hangzhou, China; ^3^ Department of Virus Inspection, Zhejiang Provincial Center for Disease Control and Prevention, Hangzhou, China

**Keywords:** vaccine, SARS-CoV-2, variants of concern, protective efficacy, COVID-19

## Abstract

The outbreak of COVID-19 (caused by SARS-CoV-2) has posed a significant threat to global public health security because of its high pathogenicity and infectivity. To date, the pathogenic mechanism of this novel coronavirus (SARS-CoV-2) is still unclear, and there is no effective treatment. As one of the most effective strategies to prevent viral infection, vaccines have become a research hotspot. Based on the current understanding of SARS-CoV-2, the research and development of its vaccines cover almost all forms of current vaccine research, including inactivated vaccines, recombinant protein vaccines, viral vector vaccines, and nucleic acid vaccines. Moreover, with the spread of the new mutant virus, it is necessary to evaluate the protection rate of previous administered vaccines. This article reviews the candidate targets, vaccine types, research and development status, progress of SARS-CoV-2 vaccines, and the effectiveness of neutralizing antibodies against SARS-CoV-2 mutants (B.1.1.7, B.1.351, P.1, B.1.617.2, and B.1.1.529) induced by these vaccines, to provide a reference for follow-up research and prevention.

## Introduction

Coronaviruses are a large group of viruses that are widespread in nature, which cause common colds to more severe diseases, such as severe acute respiratory syndrome (SARS) and the Middle East respiratory syndrome (MERS) in people and animals (1). A novel coronavirus (SARS-CoV-2) is the third known zoonotic coronavirus, and infected people often develop symptoms such as fever, cough, and fatigue, leading to severe acute respiratory infections—even acute respiratory distress syndrome (2). In 2020, the Coronavirus disease 2019 (COVID-19) epidemic occurred globally, posing a threat to public safety (3). Since the beginning of the outbreak, COVID-19 has spread rapidly. To date, it has spread to more than 210 countries and regions, causing great harm to public health and inducing devastating social and economic effects around the world (4). The World Health Organization (WHO) has declared a state of public health emergency (5–7). Given the severity of the COVID-19 epidemic, developing a safe and effective vaccine against SARS-CoV-2 infection has become a research hotspot (8). Based on the research of SARS coronavirus (SARS-CoV) and MERS coronavirus (MERS-CoV), the development of the SARS-CoV-2 vaccines has made a significant breakthrough (9, 10). However, due to the limitations of various conditions, the research and development of SARS-CoV-2 vaccines still face many challenges. It is difficult to develop a COVID-19 vaccine that can be used in people to fulfill the present demands, and its safety and efficiency need to be further proven, such as the mRNA vaccine, which was developed and administered for the first time. In addition, the protection of vaccines has been severely compromised by the constant evolution and mutation of the virus. At present, the emergence of novel SARS-CoV-2 mutations has raised concerns about the reduced sensitivity of neutralizing antibodies induced by the current vaccines (11). For instance, the Delta mutant discovered in October 2020 in India, with its strong transmissibility and basic features of immune escape, has gradually become the most important strain in new cases around the world, reducing the efficacy of immunizations significantly (12, 13); the new South African variety C.1.2, with 41.8 mutations, may be more infectious than the others.

This review mainly introduces the design targets of SARS-CoV-2 vaccines, the types, advantages, and disadvantages of vaccines being developed, the protection rate of existing vaccines against mutants, and the challenges encountered in vaccine production and prospects for the future.

## Candidate Targets for SARS-CoV-2 Vaccines

At present, seven known coronaviruses can infect humans: 229e, OC43, NL63, HKU1, SARS-CoV, MERS-CoV, and SARS-CoV-2. The first four only cause mild respiratory diseases, while the other three are β-coronaviruses, which can cause zoonosis and severe respiratory syndrome (14–16). Coronavirus is a giant single positive-strand RNA virus. Its genome is 27–32 kb (17), which mainly encodes four structural proteins: The spike protein (S), nucleocapsid protein (N), membrane protein (M), and envelope protein (E). The S protein forms spikes comprising a trimer structure on the virus’s surface, which is responsible for recognizing and binding receptors on the surface of host cells, thus mediating the adsorption and fusion of the virus and host cells. The N protein forms a spiral capsid located in the virus membrane to protect the virus RNA. The M and E proteins are essential components of the virus envelope and play an essential role in virus assembly (18, 19). Among these four structural proteins, the primary mediator of virus entry into host cells is the S protein. The S protein on the uses angiotensin-converting enzyme 2 (ACE2) as a receptor to enter the cell to cause infection and promote the virus reproduction and disease (20). Therefore, the distribution of ACE2 receptors in the human body and their affinity for the S protein determine the virulence and transmission intensity of the virus. The S protein is a type I transmembrane glycoprotein, which is composed of S1 and 2 subunits, the receptor-binding domain (RBD) of the S1 subunit is related to the recognition and binding of the host, and the S2 subunit is related to the fusion between the viral envelope and the host cell membrane. In addition, the S protein carries B cells epitopes that can induce the body to produce neutralizing antibodies and provide immune protection. The S protein is involved in viral infection and is responsible for inducing the host’s immune response (19). Therefore, it is considered a key target for vaccine design. According to current research, most of the mutation sites in the existing mutant strains are related to the S protein. The N protein of the coronavirus is necessary for the synthesis of viral RNA. It binds to the viral RNA to form a nucleocapsid, which participates in the assembly of the virus and RNA transcription simultaneously as the virus infects host cells. However, it also proves that the N protein might be a key target for vaccine design. Using certain technologies and methods, researchers quickly identified certain gene epitopes of SARS-CoV-2 (21, 22). These studies found that SARS-CoV is highly similar to SARS-CoV-2 in the gene epitopes of some T cells and B cells. If these epitopes are used as targets for vaccine design, it might be possible to form cross-protection against SARS and COVID-19, or against future mutants.

## Vaccine Development

**Figure 1:**
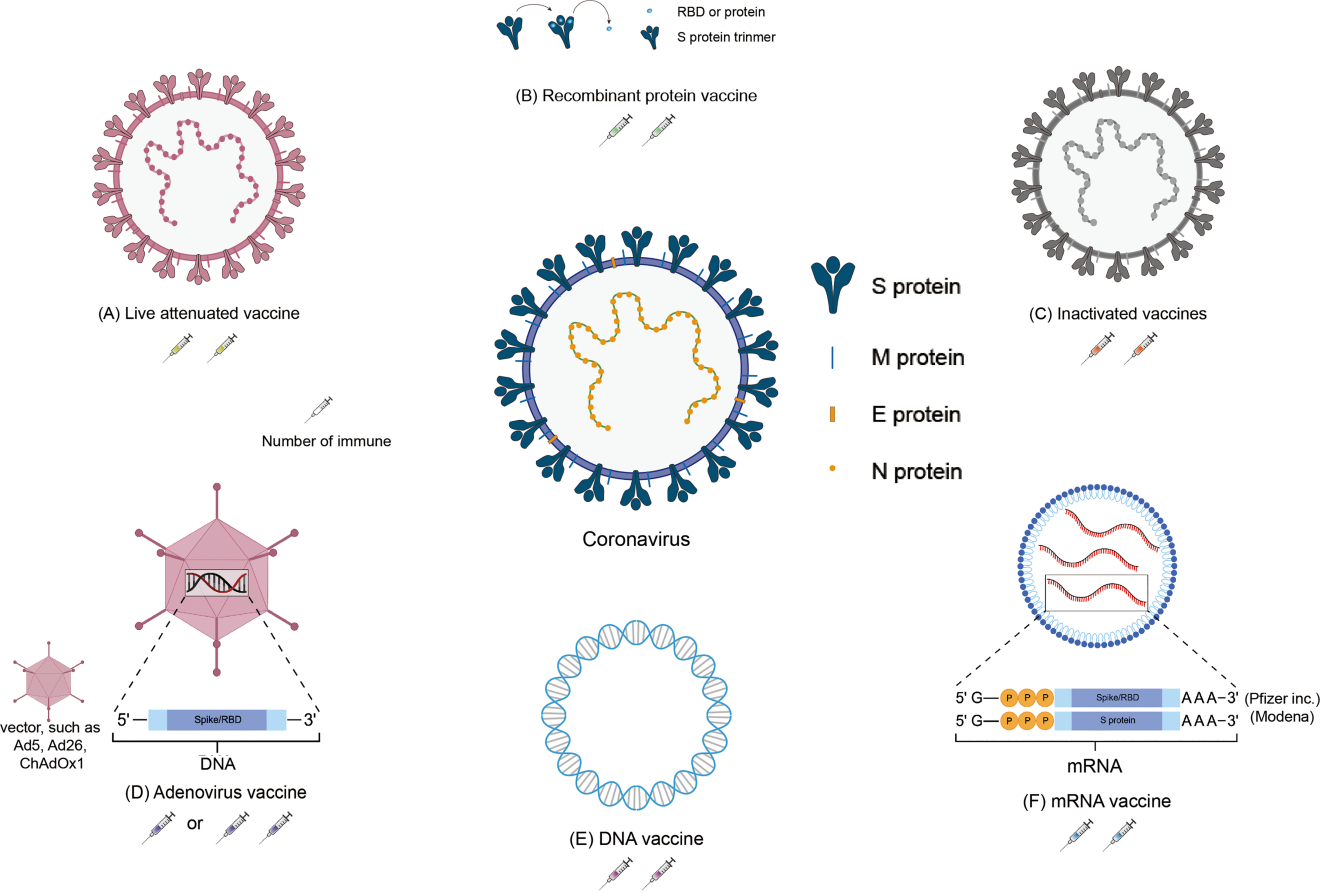
Schematic representation of the various SARS-CoV-2 vaccines. Located in the middle of the picture is the structure of coronavirus. The structure of the vaccines and the number of immunizations: **(A)** Inactivated vaccine; **(B)** Recombinant protein vaccine; **(C)** Live attenuated vaccine; **(D)** Adenovirus vaccine; **(E)** DNA vaccine; **(F)** mRNA vaccine.

There are more than 100 global SARS-CoV-2 vaccine research and development projects. At present, there are 195 vaccine candidates in the preclinical development stage and 144 vaccine candidates in the clinical development stage (as of February 20, 2022) (https://www.who.int/publications/m/item/draft-landscape-of-covid-19-candidate-vaccines) which cover all current vaccine research methods, including inactivated vaccines, recombinant protein vaccines, viral vector vaccines, and nucleic acid vaccines (mRNA vaccines and DNA vaccines) (1). The research and development institutions and progress of various types of vaccines are shown in **Table 1** and **Figure 1**.

**Table 1:** Approved vaccine information.

Vaccine type	Research and development enterprises or institutions	Dose/interval	Location	Clinical batch number	Phase
RNA	Moderna: mRNA-1273	2/1 month	America	NCT04811664	3
		2/28 days	America	NCT04470427	3
	Pfizer/BioNTech: BNT162b2	2/21 days	America, Argentina etc.	NCT04368728	2/3
DNA	Zydus Cadila: ZyCoV-D	3/28 days	India	CTRI/2021/01/030416	3
Viral Vector	Gamaleya: Sputnik Light	2/21 days	Russian Federation	NCT04741061	3
	Gamaleya: Sputnik V	2/21 days	Russian Federation	NCT04530396	3
	Janssen (Johnson & Johnson): Ad26.COV2.S	1	South Africa	NCT04838795	3
		1	America, Argentina etc.	NCT04505722	3
		1	America, Belgium etc.	NCT04614948	3
	Oxford/AstraZeneca: AZD1222	2/28 days	America, Argentina Etc.	NCT04516746	3
		2/28 days	Brazil	NCT04536051	3
	CanSino: Ad5-nCoV	1	Russian, Argentina etc.	NCT04526990	3
Inactivated	Bharat Biotech: Covaxin	2/28 days	India	NCT04641481	3
	Kazakhstan RIBSP: QazVac	2/21 days	Kazakhstan	NCT04691908	3
	Minhai Biotechnology Co: SARS-CoV-2 Vaccine (Vero Cells)	2/28 days	China	NCT04852705	3
	Shifa Pharmed Industrial Co: COVID-19 Inactivated Vaccine	2/28 days	Iran	IRCT20201202049567N3	3
	Sinopharm (Beijing, Wuhan): BBIBP-CorV (Vero Cells)	2/21 days	United Arab Emirates	ChiCTR2000034780	3
		2/21 days	Bahrain, Egypt etc.	NCT04510207	3
	Sinovac: CoronaVac	2/14 days	Turkey	NCT04942405	3
		2/14 days	Chile	NCT04992260	3
Protein Subunit	Anhui Zhifei Longcom: ZF2001	3/28 days	China, Ecuador etc.	NCT04646590	3
	Center for Genetic Engineering and Biotechnology (CIGB): CIGB-66	2/28 days	Cuba	RPCEC00000359	3
	FBRI: EpiVacCorona	2/21 days	Russian Federation	NCT04780035	3
	Medigen: MVC-COV1901	2/28 days	Taiwan, Viet Nam	NCT04695652	3
	Novavax: NVX-CoV2373	2/21 days	Mexico, Puerto Rico etc.	NCT04611802	3
		2/21 days	United Kingdom of Great Britain and Northern Ireland	NCT04583995	3

### Inactivated Vaccines

In terms of SARS-CoV-2, as of August 27, 2021, 18 inactivated vaccines are in different stages of clinical trials. Among them, eight vaccines have been approved by at least one country. They are QazVac, BBIBP-CorV, CoronaVac, Covaxin, KoviVac. (https://covid19.trackvaccines.org/vaccines/#approved). The UAE Ministry of Health has approved the BBIBP-CorV developed by Sinopharm (Beijing, China) for the SARS-CoV-2 inactivated vaccine clinical trial (Phase III) approval on June 23, 2020, and is the world’s first international clinically approved (Phase III) SARS-CoV-2 inactivated vaccine (23). On September 14, the UAE granted the vaccine an “emergency use authorization” for medical personnel. The vaccine is safe after vaccination, and the immunization program comprises two injections. After that, all recipients in the vaccine group produced high-titer antibodies, the neutralizing antibody-positive conversion rate was 99.52%, and the vaccine’s protective efficacy against COVID-19 was 79.34% (https://www.who.int/news-room/feature-stories/detail/the-sinopharm-covid-19-vaccine-what-you-need-to-know). In addition, according to reports, in July 2020 China provided urgent approval of the limited access authorization for the CoronaVac vaccine produced by Sinovac. After the Turkish Coronavirus Scientific Advisory Committee, the CoronaVac vaccine has an effective rate of 91.25% (24). The CoronaVac vaccine in Indonesia and Brazil Phase III clinical data demonstrated its protective effect. Different statistical methods might have caused the difference in the data (25).

### Live Attenuated Vaccines

An attenuated strain of SARS-CoV-2 can be obtained by deleting some aspects of the virus or *via* codon optimization. Existing live attenuated SARS-CoV-2 vaccines are all produced through codon optimization (26–28). The vaccines developed are from Indian Immunologicals Ltd., Griffith University, Acibadem Mehmet Ali Aydinlar University, and Meissa vaccine company. COVI-VAC, developed by Codagenix Ltd., is entering phase I clinical trials (NCT04619628) in the UK (29).

### Viral Vector Vaccines

Ad5-nCoV, jointly developed by CanSina and Academician Chen Wei of the Academy of Military Medical Sciences, is the first SARS-CoV-2 vaccine to publish clinical trials results publicly. The results showed that 108 volunteers had significant cellular immune responses, and neutralizing antibodies increased significantly at 14 d after vaccination, reaching a peak at 28 d, and the specific T cell response also reached a peak at 28 days. The phase II clinical trial results showed that the geometric mean titer (GMT) method detected the two-dose groups. The GMTs of the neutralizing antibodies were 19.5 and 18.3, respectively, and induced T cell immune responses. The vaccine was preliminarily proven to have good safety and tolerability, and was approved by the Chinese military as a “special needs drug” on June 25 (30). The GamCovid-VacLyo vaccine, developed by the Gamaleya Research Institute (Russia) is the world’s first approved SARS-CoV-2 vaccine. The vaccine started phase I clinical trials in June 2020 and announced on November 11 that the vaccine has an effective rate of 92%. The protection rate is 91.4% (31). It has now been approved in eight countries, including Argentina, Chile, and China.

The Ad26.COV2.S vaccine produced by Janssen (Johnson & Johnson) launched Phase I/II and Phase III clinical trials on July 25 and September 23, respectively. AZD1222 (formerly known as ChAdOx1nCoV-19) was developed by Oxford and AstraZeneca. The results of the Phase I/II clinical trial announced on July 20, 2020, showed that all subjects produced neutralizing antibodies, which could induce higher T cell immunity than mRNA vaccines and no serious adverse events (SAE) occurred. On August 18, the vaccine was registered for phase III clinical trials, and phase II/III clinical trials (NCT04400838), and phase III clinical trials were carried out in the United Kingdom, Brazil, and South Africa. Clinical data showed that the vaccine’s effectiveness in preventing COVID-19 was about 70.4%. Among people who received two high-dose vaccines (with an interval of 28 days), the effectiveness of the vaccine was about 62% (32, 33). According to early results, the effectiveness of the vaccine was about 90% for patients who received half the dose and then the full dose. However, in clinical trials, taking half-dose drugs is unreasonable, and some scientists question whether these early results are representative. Although the adenoviral vector used by AstraZeneca is non-human, to a certain extent, it avoids possible carrier neutralizing antibodies; however, the results of animal experiments are unsatisfactory, and enhancers may need to be added. It has now been approved for use in 59 countries, including Australia, Austria, and Bahrain. The nasal spray influenza virus vector new coronary pneumonia vaccine jointly developed by Xiamen University, Hong Kong University, and Beijing Wantai Biological Company has also begun clinical trials (34).

### Virus-Like Particle (VLP) Vaccines

At present, six VLP vaccines have been developed, including SpyBiotech-sponsored RBD SARS-CoV-2 HBsAg VLP vaccine at stage II/I (ACTRN12620000817943) and a Plant-based VLP vaccine at stage III/I (NCT04636697), which comprises a plant-based system that uses tobacco plants to produce VLPs (https://www.medicago.com/en/pipeline/) (35). Based on the RBD domain of SARS-CoV-2 combined with HbsAgVLPs, the RBD-HBsAg-VLPs-COVID vaccine was prepared. Adult volunteers will receive two doses at an interval of 28 days (ACTRN12620000817943) (36).

### DNA Vaccines

The Indian pharmaceutical company Zydus Cadila cooperated with the Indian Biotechnology Department to develop the world’s first new DNA crown vaccine, ZyCoV-D. The clinical trial results demonstrated a protective power of 66% and it can effectively protect against the Delta mutant strain (37). The Indian government approved it on August 20, 2021. Emergency Use authorization applies to teenagers and adults aged 12 or above. The ZyCoV-D vaccine uses a part of the DNA of the new coronavirus to produce the spike protein after entering the human body to stimulate an immune response and resist virus invasion. The advantage is that it is safer than adenovirus vector vaccines (such as AZ) and better than mRNA vaccines (such as Pfizer, Modena). The vaccine is stable and can be stored 2–8°C. The price is relatively low; however, some scientists worry that DNA vaccines might not produce high-level and lasting immune responses in the human body (38, 39). At the same time, AG0302-COVID19 produced by AnGes and INO-4800 produced by Inovio have also entered Phase III clinical trials.

### mRNA Vaccines

There are now 25 mRNA vaccines in different stages of clinical trials (https://covid19.trackvaccines.org/vaccines/#phase-3). Among them, six vaccines have been approved by at least one country. The mRNA-1273 RNA vaccine produced by Moderna officially published phase I clinical trial results on July 14, 2020. showing high titers of neutralizing antibodies in the high-dose group (100 μg, phase III clinical dose). The induced neutralizing antibody titer is four times that of the recovered patient’s plasma. At the same time, T cell immunity is induced, mainly a Th1 immune response, and no serious adverse events occur. According to its phase III clinical interim analysis data, its effective rate is 94.1% (40). The German BioNTech company, the U.S. Pfizer company, and the Chinese Fosun Pharma (FosunPharma) Group jointly develop the mRNA vaccine BNT162b2. Phase II/III clinical trials were launched on July 27, 2020, recruiting 30,000 subjects. On September 12, it was announced that the clinical trial was expanded to 43,000 subjects. In October, it was approved to conduct trials on children under 12 years of age. This was the first trial in the United States. According to the data of its phase III clinical interim analysis, the vaccine’s protection rate against COVID-19 is 95.0% (41). So far, the vaccine has been approved for use in 98 countries, including Albania, Argentina, and Australia. However, the vaccine requires cold chain transportation, which somewhat restricts its use.

### Recombinant Protein Vaccines

Recombinant protein vaccines are vaccines that use SARS-CoV-2 protein or protein fragments to stimulate an immune response. They are based on synthetic peptides or recombinant proteins. These recombinant proteins are produced in different systems, including insect cells (NCT04522089) and mammalian cells (CHO Cells, NCT04466085), yeast (42), or plants (35). At present, most recombinant protein vaccines are still in the preclinical stage, and four of them have been approved by at least one country. The NVX-CoV-2373 vaccine developed by Novavax began in September 2020, the first phase III clinical trial will be launched in the United Kingdom, and it is planned to recruit 15,000 subjects. At the end of November 2020, the United States will start phase III clinical trials with more subjects. Participants will be tested on days 0 and 21, respectively. Participants received one injection every day, and the results showed that the vaccine was well-tolerated, and no adverse events being reported under all treatment conditions (43). The ZF2001 vaccine is based on the previous MERS coronavirus spike protein (S) receptor-binding domain (RBD) (44). The concept of aggregation is to design the new coronavirus RBD in tandem repeats into a dimer (RBD-dimer) antigen, which successfully retains the vaccine’s efficacy. The neutralizing antibody titer after immunization of mice was higher than that of monomer immunity (44), and no serious adverse events related to vaccines occurred (45). After two doses of vaccine, 76% of people can produce neutralizing antibodies. After three doses of vaccine, 97% of people can produce neutralizing antibodies (44). The GMT of the antibody reached 102.5 (46), and the neutralizing GMT induced by ZF2001 was twice that of the convalescent samples, showing good immunogenicity (47). In addition, the vaccine can also produce a moderate and balanced Th1/Th2 cellular immune response.

## Methods to Detect Neutralizing Antibodies

Microneutralization assay (MNA), the plaque reduction neutralization test (PRNT), and the pseudovirus neutralization test (PVNT) are the main methods used to detect the neutralization antibody titer (48). (See **Table 2**). Before all neutralizing antibody titer tests, serum samples should be incubated for 30 min at 56°C to inactivate complement proteins, and the test can begin after balancing to room temperature.

**Table 2:** The vaccine effectiveness (VE) of various types of vaccines on different mutants.

Vaccine name	mRNA-1273	BNT162b	AZD1222	Ad26.COV2.S	NVX-CoV-2373	ZF2001	BBIBP-CorV	CoronaVac
Vaccine type	mRNA	mRNA	Adenovirus vector	Adenovirus vector	Recombinant protein	Recombinant protein	Inactivation	Inactivation
Effectiveness of non-mutant strain	94.1% (40)	95% (41)	70.4% (32, 33)	72% (49)	95.6% (50)	97% (44)	79.34% (51)	50.70% (52)
B.1.1.7 (Alpha) mutant availability	+1.6 times (2 doses, IC50, WT, MNA) (53, 54)	+1.15 times (2 doses, PRNT50, WT, PRNT) (53, 55)	48.7% (1 dose) (56)	-	85.6% (2 doses) (50)	-1.0 times (3 doses, pVNT50, D614G, pVNT) (57)	-1.4 times (2 doses, pVNT50, WT, PRNT) (58)	-0.5 times (2 doses, PRNT50, WT, PRNT) (59)
	-1.2 times (2 doses, ID50, D614G, pVNT) (60)	+2.17 times (2 doses, PRNT50, D614G, PRNT) (53, 61)	74.5% (2 doses) (56)	-	-	+1.1 times (3 doses, pVNT50, WT, pVNT) (57)	-1.3 times (3 dose, pVNT50,WT,pVNT) (62) -	
						+1.2 times (3 dose,4-6 Mo, pVNT50,WT,pVNT) (62)	-	-
B.1.351 (Beta) mutant availability	-12.4 times (2 doses, IC50, WT, MNA) (53, 54)	-2.74 times (2 doses, PRNT50, WT, PRNT) (53, 63)	10.4% (1 dose) (59)	68.1% (1 dose, ≥ 14 days, Moderate) (64)	60% (2 doses) (50)	-2.1 times (3 doses, pVNT50, D614G, pVNT) (57)	-0.4 times (2 doses, pVNT50, WT, PRNT) (58)	-0.3 times (2 doses, PRNT50, WT, PRNT) (58)
	-3.8 times (2 doses, IC50, D614G, pVNT) (65)	-3.34 times (2 doses, PRNT50, D614G, PRNT) (53, 61)	-	87.6% (1 dose, ≥ 14 days, Moderate) (64)	60.1%(2 dose) (66)	+1.9 times (3 doses, pVNT50, WT, pVNT) (57)	-2.2 times (3 dose, pVNT50,WT,pVNT) (62)	
	85% (2does) (66)	-10.3 times (2 doses, IC50, WT, MNA) (53, 54)	-	64.0% (1 dose, ≥ 28 days, Severe) (64)	-14.5 times (2 doses, ID50, D614G, pVNT) (67)	+1.1 times (3 dose, pVNT50,WT,pVNT) (62)	-	-4.4 times (2 dose, MN titer, WT, Live virus MN assay) (68)
	-	-3.4 times (2 doses, IC50, D614G, pVNT) (65)	-	81.7% (1 dose, ≥ 28 days, Severe) (64)	-6.8 times (2 doses, ID80, D614G, pVNT) (67)	-	-	-44% (2 dose) (68)
				57%(1 dose) (66)				
P.1 (Gamma) mutant availability	-4.8 times (2 doses, IC50, WT, MNA) (53, 69)	-3.8 times (2 doses, IC50, WT, MNA) (53, 69)	-	66.2% (1 dose, ≥ 14 days, Moderate) (64)	-	-1.7 times (3 doses, pVNT50, D614G, pVNT) (57)	-	50.7% (2 doses) (70)
	-3.2 times (2 doses, ID50, D614G, pVNT) (60)		-	81.9% (1 dose, ≥ 14 days, Severe) (64)	-	-1.5 times (3 doses, pVNT50, WT, pVNT) (57)	-	-
	-	-	-	52.0% (1 dose, ≥ 28 days, Moderate) (64)	-	-	-	-
	-	-	-	73.1% (1 dose, ≥ 28 days, Severe) (64)	-	-	-	
B.1.617.2 (Delta) mutant availability	-2.1 times (2 doses, IC50, D614G, pVNT) (60)	30.7% (1 dose) (56)	30.7% (1 dose) (56)	-	-	-1.4 times (3 doses, pVNT50, D614G, pVNT) (57)	95% (2 dose, ≥14 days) (71)	–2.1(2 dose, MN titer, WT,Live virus MN assay) (68)
	-	93.7% (2 doses) (56)	67.0% (2 doses) (56)	-	-	-1.2 times (3 doses, pVNT50, WT, pVNT) (57)	62% (Particle vaccination) (71)	–3.3 times (3 does, NT50. WT, pVNT) (72)
	-	-8.4 times (2 doses, ID50, D614G, pVNT) (60)	-	-	-	+1.3 times (3 dose, pVNT50,WT,pVNT) (62)	-1.7 times (3 dose, pVNT50,WT,pVNT) (62)	-

*1 dose, The vaccine was immunized once. 2 doses, The vaccine was immunized twice. 3 doses, The vaccine was immunized third. Booster dose, Vaccines to strengthen the needle. IC50, 50% true virus neutralization titer; ID50, Infection dose 50. pVNT50, 50% pseudovirus neutralization tite. PRNT50, 50% plaque reduction neutralization test. D614G, Taking the D614G mutant as the reference object. WT, Take the wild-type (Wuhan-hu-1) as the reference. Microneutralization assay (Pink), Pseudo virus neutralization test (Blue), Plaque Reduction Neutralization Test (Green), Live virus MN assay (Yellow), Effectiveness of Vaccine (Purple). 14 days, 14 days after Administration. 28 days, 28 Days after administration. Particle vaccination, >14 days for individuals who received only one dose and <14 days for individuals who received two doses. Moderate, Moderate to severe–critical Covid-19; Severe, Severe–critical Covid-19. 48.7% (1 dose), 74.5% (2 doses), 85.6% (2 doses), 10.4% (1 dose), 60% (2 doses), 50.7% (2 doses), 30.7% (1 dose), 93.7% (2 doses), 67.0% (2 doses) indicate the effectiveness of different types of SARS-CoV-2 vaccine against the different mutants. 94%, 95%, 76%, 72%, 95.6%, 97%, 79.34%, 50.70% indicate the effectiveness of different types of SARS-CoV-2 vaccine against the original variant of SARS-CoV-2.

### Microneutralization Assay, MNA

MNA is an improvement of serum virus neutralization test and is a serological test used to detect the existence of functional systemic antibodies to prevent virus infectivity (81). However, when an infectious virus is mixed with a serum antibody, the infectivity can be neutralized if the antibody binds to a closed epitope on the virus. The neutralization effect can be demonstrated by inoculating sensitive cells or organisms with antibody-virus mixtures. Detection is carried out with a constant number of viruses and continuously diluted serum samples until virus neutralization is no longer detected. The neutralizing antibody titer is the reciprocal of the final dilution multiple of the serum with neutralizing activity. The data described in this review using the MNA method are expressed as the IC50 and MN50.

### Plaque Reduction Neutralization Test, PRNT

PRNT is currently one of the laboratory standards for monitoring neutralizing antibodies and needs to be tested with the virus. PRNT counts virus plaques and calculates the PRNT endpoint titer at each selected percentage decrease in virus activity (usually 50 or 90 percent). As a gold standard, the disadvantage of PRNT is that it is labor-intensive and difficult to adapt to high-throughput; therefore, it is difficult to use it for large-scale surveillance or in vaccine trials. Studies have found that the PRNT IC90 is more sensitive than MNA to detect antibodies (82). Moreover, because of the high pathogenicity and infectivity of SARS-COV-2, all tests related to the use of live viruses must be carried out in a biosafety level III (BSL-3) laboratory. The neutralization titer is calculated as the reciprocal of the highest serum dilution that reduces plaque formation by 50%. The data calculated using the PRNT method are expressed as PRNT50.

### Pseudo Virus Neutralization Test, PVNT

PVNT replaced the envelope protein of lentivirus (or vesicular stomatitis virus VSV) with novel coronavirus’s Spike protein to simulate novel coronavirus infection. The false virus has no complete live virus components and can only be replicated in one round. Compared with PRNT and MRNT, PVNT is a sensitive, accurate, and reproducible method. The SARS-CoV-2 pseudovirus takes the replication-defective HIV-1 virus as the skeleton, encapsulates the surface spike glycoprotein of SARS-CoV-2, binds to the human angiotensin-converting enzyme 2 (ACE2) receptor through the Spike protein receptor-binding domain (RBD), and infects the cells expressing human ACE2, simulating the process of virus invasion. Since pseudoviruses can only be infected once and cannot be replicated, they can be used in biosafety level 2 (BSL-2) laboratories.

## Efficacy of Approved Vaccines Against Mutant Strains

**Figure 2:**
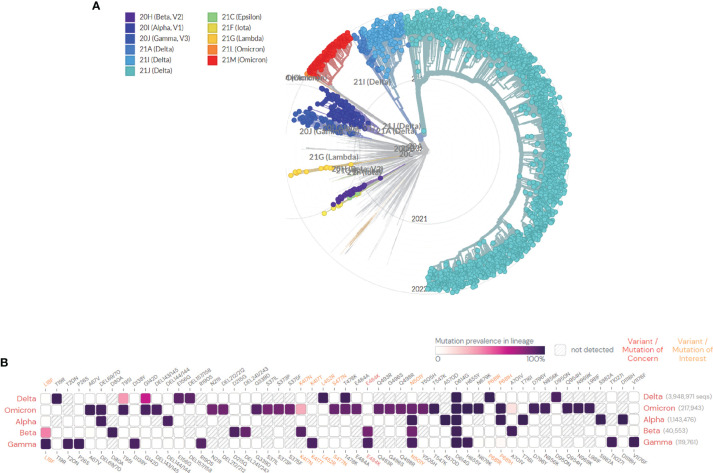
**(A)** SARS-CoV-2 global phylogeny reveals the Omicron lineage. The Nextstrain platform provides a time-calibrated worldwide SARS-CoV-2 phylogeny (https://nextstrain.org/ncov/gisaid/global). Red and orange indicate the current status of the highly observed Omicron variant, and the variants of concern (VOCs) (Alpha, Beta, Gamma, Delta, and Omicron) and variants of interest (VOIs) (Lambda, Lota, and Epsilon) are colored as indicated. These variants are all described in detail in the article. **(B)** Mutation prevalence across lineages. The Outbreak platform provides S protein mutation prevalence across lineages. (Mutations with > 75% prevalence in at least one lineage) (https://outbreak.info). The figure describes in detail the mutation prevalence in lineage of VOCs. Orange indicates a mutation of interest and the red indicates a mutation of concern. Blank indicates that the mutation has not detected. White to purple indicates the prevalence of the mutation in all sequences.

**Figure 3:**
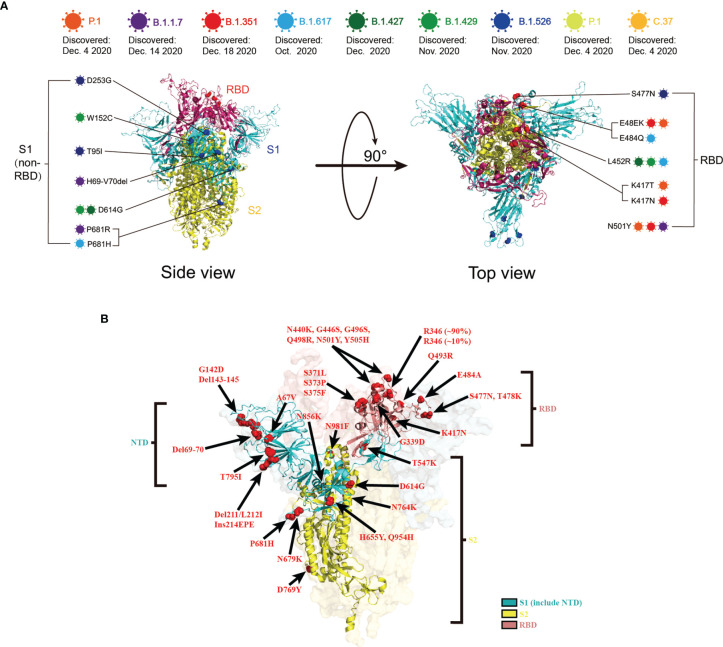
**(A)** Schematic representation of the mutation sites on various mutant viruses. Schematic diagram of the SARS-CoV-2 spike protein structure and the mutation sites of the mutant strains in this review: B.1.1.7 (Purple), B.1.351 (red), P.1 (orange), B.1.617 (blue), B.1.427 (dark green), B.1.429 (light green), B.1.526 (dark blue), AY.1 (yellow), C.37 (light orange). **(B)** Mutations within B.1.1.529 denoted on the full SARS-CoV-2 spike trimer. The SARS-CoV-2 spike structure was downloaded from PDB 7DZW.

The RNA virus to which the new coronavirus belongs has a very high mutation rate, promoting its transmission and toxicity, and allowing variants to escape immunity from previous infections or vaccinations (83). Many studies on different vaccines against new coronavirus mutant strains have proved that vaccines have different protective immune responses against different new coronavirus mutant strains, i.e., cross-protection. It is generally believed that virus strains of different genotypes or subtypes have cross-protective effects. According to research, the protective effect of the same vaccine on different mutants of the new coronavirus is mostly showing a downward trend, but it still has a protective effect. According to the International Health Organization, the main mutant strains of the new coronavirus are now identified as: B.1.1.7, B.1.351, P.1, B.1.617.2, and B.1.1.529. Five mutant strains of C.37 were first found in the United Kingdom, South Africa, Brazil, California, India, New York, India, and Peru, where they became widespread (4). The protective efficiency of existing vaccines against mutant strains is shown in **Tables 2**, **3**. We constructed a radial ring from SARS-CoV-2 sequences available in the GISAID EpiCoV collection using a subsampling technique. To provide context for the isolated viral genome, we conducted a targeted phylogenetic analysis utilizing all variants samples posted to GISAID on January 1, 2022, as a baseline (**Figure 2A**). We also analyzed a lineage comparison map with a large number of sequences from GISAID to understand the mutations with high prevalence (**Figure 2B**).To better characterize the mutational context responsible for the above nine mutants’ neutralization resistance, we collect the contribution of mutations located both within and outside of the RBD region of the spike protein (See **Figures 3A, B**).

**Table 3:** The vaccine effectiveness (VE) of different types of vaccines and serums on the omicron variant.

Vaccine/Serum name	mRNA-1273	BNT162b	AZD1222	Ad26.COV2.S	CoronaVac	BBIBP-CorV	ZF2001
Vaccine/Serum type	mRNA	mRNA	Adenovirus vector	Adenovirus vector	Inactivation	Inactivation	Adenovirus vector
B.1.1.529(Omicron)mutant availability	-8.6 times (2 dose, ID50, D614G, pVNT) (73)	-22 times (2 dose, FRNT50, D614G, aVNT) (74)	-1.6 times (2 dose, ID50, D614G, pVNT) (73)	-2.5 times (2 dose, ID50, D614G, pVNT) (73)	-4.3 times (2 dose, MN titer, WT, Live virus MN assay) (68)	-5.1 times (3 dose, pVNT50,WT,pVNT) (62)	-4.8 times (3 dose,4-6 Mo, pVNT50,WT,pVNT) (62)
	-6.5 times (booster dose, ID50, D614G, pVNT) (73)	-21 times (2 dose, ID50, D614G, pVNT) (73)	-21 times (2 dose, ID50, WT, pVNT) (75)	-13 times (booster dose, GMNT, D614G, pVNT) (76)	-40 times (3 does, NT50, WT, pVNT) (72)		
	-26.6 times (2 dose, NT50, D614G, pVNT) (77)	-6.5 times (booster dose, ID50, D614G, pVNT) (73)	-12.7 times (2 dose, FRNT50, Victoria, aVNT) (78)		-16.5 (3 does, NT50, WT, pVNT) (72)		
	-5.1 times (booster dose, NT50, D614G, pVNT) (77)	-14.5 times (2 dose, NT50, D614G, pVNT) (77)	-3.6 times (2 dose, FRNT50, Delta, aVNT) (78)				
	-43 times (2 dose, <3 mo, GMNT, D614G, pVNT) (76)	-5.0 times (booster dose, NT50, D614G, pVNT) (77)					
	-6 times (booster dose, GMNT, D614G, pVNT) (76)	-43 times (2 dose, <3 Mo, GMNT, D614G, pVNT) (76)					
	-39 times (2 dose, ID50, WT, pVNT) (75)	-6 times (booster dose, GMNT, D614G, pVNT) (76)					
	-42 times (2 dose, ID50, WT, multi-cycle microneutralization assay) (79)	-37 times (2 dose, ID50, WT, pVNT) (75)					
	-16.7 times (booster dose, ID50, WT, multi-cycle microneutralization assay) (79)	-23 times (2 dose, ID50, WT, multi-cycle microneutralization assay) (79)					
		-7.5 times (booster dose, ID50, WT, multi-cycle microneutralization assay) (79)					
		-14.2 times (2 dose, FRNT50, Victoria, aVNT) (78)					
		-3.6 times (2 dose, FRNT50, Delta, aVNT) (78)					
		-34 times (2 dose, NT50, B.1, pVNT) (80)					
		-12 times (2 dose, NT50, Delta, pVNT) (80)					
		-8 times (booster dose, NT50, B.1, pVNT) (80)					
		-2 times (booster dose, NT50,Delta, pVNT) (80)					
	-5.9 times (2 dose, ID50, D614G, aVNT) (73)						
	-4.1 times ((booster dose, ID50, D614G, aVNT) (73)						

*1 dose, The vaccine was immunized once. 2 doses, The vaccine was immunized twice. 3 doses, The vaccine was immunized thrice. Booster dose, Vaccines to strengthen the needle. ID50, Infection dose 50. NT50, 50% neutral titer. GMNT, geometric mean neutralization titer. FRNT50, 50% focus reduction neutralization test value. MN titer, mean neutralization titer. D614G, Taking the D614G mutant as the reference object. WT, Take the wild-type (Wuhan-hu-1) as the reference object. Victoria, Take the Victoria as the reference object. B.1, Taking the B.1 as the reference object. Delta, Taking the Delta as the reference object. Pseudo virus neutralization test (Blue). Authentic virus neutralization tesrat (Orange); multi-cycle microneutralization assay (Red).

### B.1.1.7 (Alpha) Mutant

B.1.1.7 mutant strains were first discovered in the UK in December 2020, starting in mid-January 2021, and peaking in the first week of March. On the 18th of the same month, it was renamed VOC-202012/01. In early February 2021, the variants of the new coronavirus discovered in the United Kingdom and the United States first appeared as a combination, which is easy to spread and resistant to drugs (4). Among the nucleic acid vaccines, studies have found that the BNT162b2 vaccine produced by BioNTech, Pfizer, and Fosun Pharma will be effective against infection with any documented B.1.1.7 variant at 14 days or more after the second injection. The estimated validity is 89.5% [95% confidence interval (CI), 85.9–92.3. Using a cohort study design, the vaccine’s effectiveness was evaluated by comparing the incidence of infection among vaccinated persons with the infection rate of the antibody-negative national cohort. The effectiveness against the B.1.1.7 variant was estimated as 87.0% (95% CI, 81.8–90.7) (84). In another study, samples were collected between 7 and 91 days after the second dose of the BNT162b2 vaccine. In this study, a live virus neutralization test was used, and the 50% neutralization titer determined using PRNT was 1.15 times higher than that against the wild-type strain and 2.17 times higher than that against D614G. The nucleic acid vaccine mRNA1273RNA, produced by Moderna, has the highest sensitivity to the mutant strain. In a study using a live virus neutralization test, samples obtained between 7 and 180 days after the second dose of the vaccine were 50% more sensitive. The 50% neutralization titer determined using PRNT was 1.6 times higher than that against the wild-type strain (53–55, 61). Another study found that the neutralizing titer of mRNA-1273 against B.1.1.7 was 1.2 times lower than that against D614G (60). For the viral vector vaccines, studies have found that the effectiveness of one dose of the vaccine OxfordChAdOx1-S (AZD1222), produced by Oxford and AstraZeneca, is 48.7% (95% CI, 45.5–51.7). The effectiveness of two vaccine doses was 74.5% (95% CI, 68.4–79.4) (56). Among recombinant protein vaccines, studies have found that the protection rate of the NVX-CoV2373 vaccine produced by Novavax against this variant is 85.6% (50). For the inactivated vaccine, the CoronaVac produced by Sinovac showed a significant decrease in B.1.1.7 serum neutralization GMTs by 0.5 times (95% CI, 0.3–0.7). The BBIBP-CorV (Vero Cells) vaccine, produced by Sinopharm, showed a significant decrease in B.1.1.7 serum neutralization GMTs by 1.4 times (95% CI, 0.9–2.2) (58). Among the recombinant protein vaccines, the ZF2001 vaccine produced by Anhui Zhifei Longcom showed that the neutralization titer of the vaccine against the B.1.1.7 mutant was 1.1 times higher than that against the wild-type strain (Wuhan-1 Reference strain). Compared with the reference strain (D614G mutant), the neutralization titer was reduced by 1.0 times (57).

### B.1.351(Beta) Mutant

B.1.351 was found in South Africa in December 2020. On May 31, 2021, B.1.351 was renamed the Beta variant virus (https://nextstrain.org/ncov/gisaid/global). For nucleic acid vaccines, studies have shown that the BNT162b2 vaccine is 75.0% (95% CI, 70.5–78.9) effective against any documented B.1.351 variant infection at 14 days or more after the second injection. A cohort study design was used to evaluate the vaccine’s effectiveness by comparing the incidence of infection among vaccinated persons with the infection rate of the antibody-negative national cohort. The effectiveness of the variant was estimated to be 72.1% (95% CI, 66.4–76.8) (84). In another study, samples were collected between 7 and 91 days after inoculation with the second dose of the BNT162b2 vaccine. In the experimental method using PRNT, the neutralization titer of the 50% live virus neutralization test was 2.74 times lower than that against the wild-type strain and 3.34 times lower than that against D614G. In the experimental method using MNA, the neutralization titer of the 50% live virus neutralization test was 2.74 times lower than that against the wild-type strain and 3.34 times lower than that against D614G. The neutralization titer of the 50% live virus neutralization test was reduced by 10.3 times. The nucleic acid vaccine mRNA-1273 RNA was the least sensitive to mutants, and samples were collected 7 to 180 days after the second vaccination. Under the experimental method using MNA, the neutralization titer of the 50% live virus neutralization test was 12.4 times lower than that against the wild-type strains (53, 54, 61, 63). It was also found that the neutralization titer of mRNA-1273 against B.1.351-v1, B.1.351-v2, and B.1.351-v3 was 6.9, 7.3, and 8.4 times lower than that against D614G, respectively (60). Among viral vector vaccines, studies have found that a dose of Oxford ChAdOx1-S (AZD1222) is 10.4% effective against this variant (95% CI, 76.8–54.8) (59). Among the recombinant protein vaccines, the protection rate of the NVX-CoV2373 vaccine against this variant was 60% (50). It was found that the geometric mean ID50 titer of serum of people receiving the NVX-CoV2373 vaccine was 14.5 times lower than that of D614G, and its GMT was slightly lower than that against the wild-type or D614G strains (59). Among the recombinant protein vaccines, the ZF2001 vaccine produced by Anhui Zhifei Longcom showed that the neutralization titer of the vaccine against the B.1.351 mutant was 1.9 times higher than that against the wild-type strain (Wuhan-1 Reference strain). Compared with the reference strain (D614G mutant), the neutralization titer was reduced by 2.1 times (57). For the inactivated vaccines, the GMTs of BBIBP-CorV also decreased by 1.6-fold, from 110.9 (95% CI, 76.7–160.2) to 70.9 (95 CI, 50.8–98.8) (45), which was significantly lower than that previously reported in convalescent plasma (more than ten times) or mRNA vaccine receptor antiserum (more than six times) (54, 85). For another inactivated CoronaVac vaccine, the GMT of the mutant was reduced by 0.3 times (95% CI, 0.2 –0.4). For the BBIBP-CorV (Vero Cells) vaccine, we observed a significant decrease of B.1.1.7 serum neutralization GMTs by 0.4 times (95% CI, 0.3–0.8) (58). Among vector vaccines, a single dose of AD26.COv2, manufactured by Johnson & Johnson, was 52.0% effective at 14 days after vaccination in patients with moderate to severe B.1.351 infection and at 28 days after vaccination, the vaccine’s effectiveness was 73.1%. In severe to critically ill patients, the vaccine was 64.0% effective at 14 days after vaccination and 81.7% effective at 28 days after vaccination (64).

### P.1 (Gamma) Mutant

On January 14, 2021, P.1 was found in the Brazilian state of Amazon. P.1 was named the Gamma Variant Virus on 31 May 2021 (https://nextstrain.org/ncov/gisaid/global). Among nucleic acid vaccines, serum sample were taken at least 7 to 32 days after the second dose of the BNT162b2 vaccines. Under the experimental method using MNA, the neutralization titer of the 50% live virus neutralization test was 3.8 times lower than that of the wild-type strain. For the nucleic acid vaccine mRNA1273RNA, serum samples taken at least 7 to 180 days after the second vaccination showed a 4.8-fold reduction in the neutralization titer in 50% of live virus neutralization tests compared with the wild-type strains under the experimental method using MNA (53, 69). The neutralization titer of mRNA-1273 against P.1 was also 3.2 times lower than that against D614G (60). Among the recombinant protein vaccines, the ZF2001 vaccine showed a neutralization titer against the P.1 mutant that was 1.5 times lower than that against the wild-type strain (Wuhan-1 Reference strain). Compared with the reference strain (D614G mutant), the neutralization titer was reduced by 1.7 times (57). Among inactivated vaccines, the primary efficacy of BBIBP-CorV (VeroCells) against symptomatic COVID-19 is 50.7% (95% CI, 36.0–62.0) (70). Among vector vaccines, a single dose of AD26.COv2 was 66.2% effective at 14 days after vaccination in patients with moderate to severe P.1, and at 28 days after vaccination, the vaccine’s effectiveness was 68.1%. In severe to critically ill patients, the vaccine was 81.9% effective at 14 days after vaccination and 87.6% effective at 28 days after vaccination (64).

### B.1.617.2 (Delta) Mutant

As of April 20, 2021, the double mutant strain B.1.617 (Delta) has been detected in more than 20 countries, although no major outbreaks similar to India have occurred in these other countries (4). Among the nucleic acid vaccines, the BNT162b2 vaccine has shown that the effectiveness after the first dose of vaccination is 30.7% (95% CI, 25.2–35.7); after the second dose of vaccination, the effectiveness is 93.7% (95% CI, 91.6–95.3) (56). In the live virus neutralization test after the second dose of vaccination, the neutralization titer of the BNT162b2 vaccine was reduced by 50% compared with the reference strain. The neutralization titer of the B.1.617.2 mutant was compared with that of the D614G mutant for the mRNA-1273 vaccine. The IC50 of the B.1.617.2 mutant was 2.1 times lower than that of the D614G mutant (60). Among adenovirus vaccines, AZD1222 was shown to be 30.7% (95% CI, 25.2–35.7) effective after the first dose, similar to BNT162b2 (56). Among the inactivated vaccines, the neutralization effect of the Covaxin vaccine produced by Bharat Biotech, India, against the B.1.617.2 mutant strain was compared with that against the original strain (D614G). For D614G vs. B.1.617.2, the GMT ratio was 1.95, (95% CI: 1.60–2.23; p-value < 0.0001) (86). Among the recombinant protein vaccines, the ZF2001 vaccine showed a neutralization titer against the B.1.617.2 mutant that was 1.2 times lower than that against the wild-type strain (Wuhan-1 Reference strain). Compared with the reference strain (D614G mutant), the neutralization titer was reduced by 1.4 times (57). For the Covishield vaccine, produced by Serum Institute of India, the GMT ratio was 3.553 (95% CI: 1.252–10.08) for D416G vs. B.1.617.2. After the second dose, the GMT ratio was 22.43 (95% CI: 10.96–45.9) (87).

### B.1.1.529 (Omicron) Mutant

The B.1.1.529 variant was first discovered in southern Africa on November 9, 2021. It was first isolated in late November 2021 and has been classified as the highest-level “closely watched variant” (VOC) by the World Health Organization (WHO), naming it Omicron. More than 38 countries have reported confirmed cases of Omicron, causing major public health concerns about its increased infectivity (88). Additionally, its spike protein contains an alarming number of mutations (>30; **Figure 3B**), at least 15 of which are in the receptor-binding domain (RBD), the primary target of neutralizing antibodies (89). At the same time, it also raises major concerns about the possibility of highly immune escape of this mutant.

Among nucleic acid vaccines, in the pseudovirus experiment, the mRNA-1273 vaccine showed a neutralization titer against the B.1.1.529 mutant that was 8.6-fold lower than that against the D614G, and a 6.5-fold decrease in ID50 was seen for a booster dose of mRNA-1273 vaccine (73). Another study showed that in the neutralization test of two doses of the nucleic acid vaccine mRNA-1273, the NT50 against B.1.1.529 was 26.6 times lower than that against D614G, and its NT50 decreased 5.1 times in the booster dose (77). In addition, the geometric mean neutralization titer (GMNT) decreased by 43-fold for people recently (< 3 months) vaccinated with mRNA-1273, and by 6-fold for those who were booster vaccinated (76). Another study showed that the neutralization of individuals inoculated with mRNA-1273 was 39 times lower than that with Wuhan-Hu-1 (75). Individuals vaccinated with two doses of mRNA-1273 had a 42-fold reduction in neutralization and a 16.7-fold decrease in neutralization with a booster shot (79).

For another nucleic acid vaccine, BNT162Bb2, a 21-fold decrease in ID50 for BNT162b2 vaccinated in the pseudovirus experiment and a 6.5-fold decrease in the booster of BNT162Bb2 compared with D614G (73). Similarly, studies have shown that compared with D614G, the NT50 of BNT162b2 vaccinated individuals decreased by 14.5 times, and the booster of BNT162Bb2 decreased by 5.0 times (77). Other studies showed a GMNT decrease of 122-fold for recently (< 3 months) for individuals vaccinated with BNT162Bb2, an a 4-fold reduction for those who are booster vaccinated (76). Another study showed that the neutralization of individuals inoculated with BNT162Bb2 was 37 times lower than Wuhan-Hu-1 (75). In another study, individuals vaccinated with two doses of BNT162Bb2 had a 23-fold reduction in neutralization and a 7.5-fold decrease in neutralization with a booster shot (79). Another study showed that individuals receiving BNT162b2 (two doses) have a 34-fold decrease in neutralization efficiency over one to three months compared with B.1 and a 12-fold decrease compared with Delta. For individuals injected with the booster, the neutralization efficiency was eight times lower than B.1 and two times lower than that of Delta (80).

In the Authentic virus experiment, the ID50 for the total dose of the two mRNA vaccines decreased by 5.9 times compared with D614G, while the ID50 for the booster shot decreased by 4.1 times (73). Another study showed the 50% focus reduction neutralization test value (FRNT50) for all patients vaccinated decreased from 1963 to 89, a 22-fold decrease (95% CI 16–30). Vaccine-only individuals (95% CI 15–32) had a 22-fold reduction in multiples (74). In another study, after 28 days of three doses, the neutralization titer of BNT162b2 against Omicron was 14.2 times lower than Victoria and 3.6 times lower than Delta. The neutralization titer of Omicron increased 2.7 times after injection of BNT162b2 booster dose (78).

Among viral vector vaccines, compared with D614G, the ID50 of injected Ad26. COV2.S decreased by 2.5 times, and injected AZD1222 decreased by 1.6 times in the pseudovirus experiment (73). Another study showed that the neutralization of individuals inoculated with AZD1222 was 21 times lower than with Wuhan-Hu-1 (75). In the Authentic virus experiment, after 28 days of three doses, the neutralization titer of ADZ1222 against Omicron was 12.7 times lower than with Victoria and 3.6 times lower than with Delta (78). However, the serum of patients inoculated with AD26.cov2.s (one dose) or Sputnik V showed little neutralizing activity (75). In addition, the GMNT decreased by 13-fold for booster vaccinated people (76).

For inactivated vaccines, the serum from patients inoculated with BBIBP-CORV also showed little neutralizing activity (75).

Studies have analyzed the neutralizing activity of convalescent serum to Omicron. One study showed that the neutralization of convalescent serum to Omicron in the pseudovirus test was at least 32 times lower than that of D614G (76). Other studies showed that the titer of neutralizing antibody to Omicron in convalescent serum of patients with early strain infection decreased by 36 times, while the titer of neutralizing antibody to Omicron in convalescent patients infected with the Delta strain decreased by 39 times (90).

Another study that collected convalescent serum for different mutants and conditions carried out a neutralization test for Omicron and compared it with the neutralization titer of α, β, Gamma, and Delta. The results showed that compared with α, the serum neutralization titer of Omicron decreased by 33.8 times (p < 0.0001), β by 11.8 times (p = 0.0001), Gamma by 3.1 times (p = 0.001), and Delta by 1.7 times (p = 0.0182). At the same time, it was confirmed that the serum of people who received the vaccine and were infected with the Delta virus had a significantly higher neutralization effect on all viruses than those who were only infected with Delta virus (78). By analyzing samples at six months and 12 months after the onset of symptoms (M6 and M12), it was found that the serum neutralization titer of Omicron was 53 and 23 times lower than that of D614G and Delta, respectively. It was also found that a single dose of vaccine could increase the cross-neutralization antibody response of previously infected individuals to Omicron (91).

As for infection after vaccination, some studies have shown that the serum GMT to Omicron of previously infected vaccinators decreased significantly (GMT of mRNA-1273 decreased by nine times, GMT of BNT162b by 12 times, and GMT of Ad26.COV2.S by 17 times), but most of them retained detectable neutralization abilities (79). Another study confirmed this view, showing that the neutralization of previously infected vaccinators to Omicron decreased by 22 times (95% CI 16–34) (78).

The above results show that Omicron shows a strong immune escape ability, which means that recovered patients are still vulnerable to infection by Omicron; however, the serum of vaccinated patients has enhanced the neutralization effect of the mutant, so vaccination is still necessary.

## Current Challenges

SARS-CoV-2 is a brand-new virus that has only just appeared in humans, and people do not understand it comprehensively. Its antigenic characteristics, variability, and immune response characteristics need to be further determined.

It remains to be seen whether vaccines can provide broad protection to the population. Immune aging means that the response of the elderly to vaccination is usually poor. Studies have shown that influenza vaccines for the elderly need specific formulations, including more antigens or adjuvants. Influenza vaccines seem to require higher neutralization titers for the elderly than do young people for SARS-CoV-2. This problem also needs to be solved urgently.

It is necessary to pay attention to the phenomenon of disease enhancement after vaccination, i.e., the antibody dependence of virus infection (ADE). Some studies have shown that in animals that are re-exposed to live viruses after immunization with SARS-CoV and MERS-CoV vaccines, disease progression might be aggravated. Although the ADE effect has not been found after SARS-CoV-2 vaccination, it still needs further research and close attention.

Before putting the vaccine into clinical trials, two critical steps need to be completed. First, the vaccine should be tested in an appropriate animal model to verify its protective effect. Animal models infected with SARS-CoV-2 might be challenging to establish, and the virus does not grow in wild-type mice and can only induce mild diseases in transgenic animals that express human ACE2. Second, the vaccine needs to be tested for animal toxicity, which must be conducted in a manner that complies with the Drug non-Clinical Research quality Management Code (GLP) and usually takes 3 to 6 months to complete.

Vaccines for human use must be produced in compliance with the Drug production quality Management Code (GMP) to ensure the continuous quality and safety of the vaccine. This requires specialized facilities, trained technicians, appropriate documentation, and raw materials for production to meet GMP standards. These processes are not perfect for many SARS-CoV-2 vaccines in the preclinical stage, and standards must be established from scratch.

The production capacity to provide sufficient quantities of GMP-quality vaccines is not yet available. It can be relatively easy to achieve for existing vaccine platforms and infrastructure, such as inactivated or live attenuated vaccines. It takes time to establish a GMP production platform for other vaccines, such as mRNA vaccines, even in developed countries in Europe and the United States. The demand for vaccines in pandemics also far exceeds the current production capacity.

Nonetheless, studies have shown that completely vaccinated persons who develop breakthrough infections have a peak viral load comparable to unprotected patients and are capable of transmitting the virus effectively in the crowd, even to fully vaccinated contacts (92). However, the overwhelming evidence indicates that vaccination seems to provide significant protection against serious illness caused by all major virus types (93).

## Conclusions

The continuous emergence of mutants of the new coronavirus has not only created marked obstacles to the prevention and control of the epidemic, but also introduced greater challenges and tests to the research and development of vaccines. From the perspective of vaccine research and development, we can consider the possibility of a multi-target combined vaccine and a breakthrough in adjuvant research to improve the effectiveness of the vaccine and prolong its half-life. For example, the Novavax Company of the United States recently launched a clinical study that is expected to a combine SARS-CoV-2 vaccine, an influenza vaccine, and adjuvants or vaccine enhancers into a universal vaccine for both influenza and COVID-19 (94). Furthermore, Public Health England has reported (80) that vaccine brands can be mixed in “extremely rare occasions”. In these circumstances, as both the vaccines are based on the spike protein, it is likely that the second dose will help to boost the response to the first dose. In addition, the development of a universal vaccine will also help people cope with future outbreaks. Several recent studies (95, 96) suggested that a “universal” vaccine that can fight novel coronavirus and new variants, and protect people from future pandemics, might be launched soon, and several companies are developing the vaccine in the “contract phase.” Its advantage is that if the novel coronavirus’s genome is sequenced and there is an obvious protein as a target, the vaccine can quickly adapt to the new virus. From the perspective of COVID 19 genomic research, researchers can also collect the genomes of known new mutated strains, determine their pedigree relationships, and build a general framework for novel coronavirus evolution. It is important to understand the law of pathogen variation, to track COVID-19’s epidemiology, and predict the direction of virus evolution. For example, given the trend that Delta strain is gradually becoming the dominant variety, it is necessary to develop a vaccine against this mutant strain.

In summary, the development of SARS-CoV-2 vaccines still faces significant challenges and requires the unremitting efforts of researchers all over the world. In addition, the development, testing, production, and distribution of vaccines in a short period requires close coordination among pharmaceutical companies, governments, regulators, and the WHO to respond to the epidemic more quickly, scientifically, and accurately.

## Author Contributions

KC and YZ conceived the article; KC, CL, and YG drafted the article; CL, YG, ZF, and HZ performed the statistical analyses, interpreted the data, and generated figures. All authors contributed substantially to the content and reviewed or edited the manuscript. All authors approved the manuscript.

## Funding

The study was supported by the Key Research and Development Program of Zhejiang Province (2021C03044), the Major horizontal project of Zhejiang Shuren University(2020D1284) and the Innovation and Entrepreneurship Training Program for College students of Zhejiang Shuren University (202011842024).

## Conflict of Interest

The authors declare that the research was conducted in the absence of any commercial or financial relationships that could be construed as a potential conflict of interest.

## Publisher’s Note

All claims expressed in this article are solely those of the authors and do not necessarily represent those of their affiliated organizations, or those of the publisher, the editors and the reviewers. Any product that may be evaluated in this article, or claim that may be made by its manufacturer, is not guaranteed or endorsed by the publisher.
